# More than an infection: the ecological puzzle of recurrent urinary tract infections

**DOI:** 10.3389/fcimb.2026.1927507

**Published:** 2026-09-02

**Authors:** Layla Musleh, Marco Montilli, Maria Grazia Ammendolia, Alessandro Sciarra, Anna Riccioli, Linda Maurizi, Catia Longhi

**Affiliations:** 1 Department of Public Health and Infectious Diseases, Sapienza University of Rome, Rome, Italy; 2 Department of Pediatric Surgery, San Camillo-Forlanini Hospital, Rome, Italy; 3 Faculty of Medicine and Dentistry, Sapienza University of Rome, Rome, Italy; 4 National Center of Artificial Intelligence and Innovative Technologies for Health, Istituto Superiore di Sanità, Rome, Italy; 5 Department of Maternal Infant and Urologic Sciences, Policlinico Umberto I Hospital, Sapienza University of Rome, Rome, Italy; 6 Department of Anatomy, Histology, Forensic Medicine and Orthopaedics, Unit of Histology and Medical Embryology and Unit of Human Anatomy, Sapienza University of Rome, Rome, Italy

**Keywords:** antimicrobial therapy, host microbe interactions, microbial persistence, mucosal immunity, recurrent urinary tract infections, urinary microbiome

## Abstract

Recurrent urinary tract infections (rUTIs) represent one of the most common infectious conditions worldwide, yet their pathophysiology extends far beyond repeated episodes of acute bacterial cystitis. Increasing evidence indicates that recurrence may arise through overlapping mechanisms including reinfection from intestinal or periurethral reservoirs, intracellular bacterial persistence, microbial dysbiosis, impaired mucosal immunity and chronic inflammatory remodelling of the bladder microenvironment. Current diagnostic frameworks remain largely based on symptom-based definitions and standard urine culture, approaches that incompletely capture the biological complexity of recurrent disease. This limitation is evident even at the definitional level, where clinically pragmatic categories often fail to reflect the heterogeneous mechanisms underlying recurrence. Advances in expanded urine culture techniques, metagenomics and metabolomics have reshaped the understanding of the urinary tract as a dynamic ecological system interconnected with vaginal, intestinal and prostatic microbial compartments. These approaches have identified diverse microbial communities, virulence-associated functional profiles and host-microbe interactions linked to recurrence-prone phenotypes. Significant challenges continue to persist in elucidating the biological mechanisms driving recurrence. Addressing these gaps is essential to improve disease characterization and support the development of more effective diagnostic and therapeutic approaches.

## 1 Introduction

**Figure 1:**
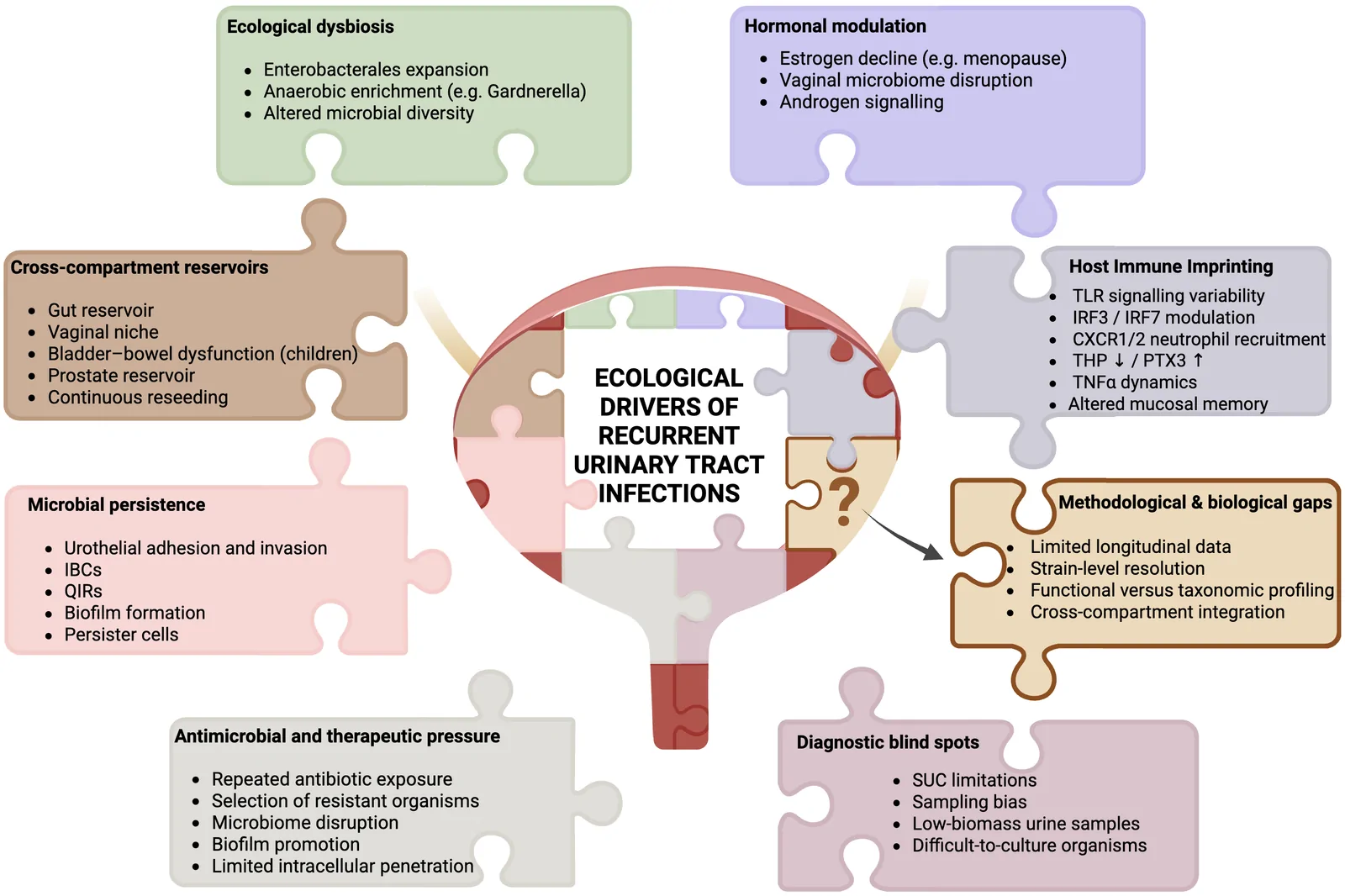
The ecological drivers of recurrent urinary tract infections. Schematic overview of the interconnected factors contributing to recurrent urinary tract infections (rUTIs). BPH, benign prostatic hypertrophy. Created in BioRender https://BioRender.com/r8evll4.

Urinary tract infections (UTIs) are among the most common infectious diseases worldwide (Foxman et al., 2000; Yang et al., 2022). Although the vast majority are caused by bacteria, viral and fungal pathogens and, more rarely, parasites may also cause urinary tract infections (Mancuso et al., 2023; ElShewy, 2024). However, bacteria are responsible for the overwhelming majority of cases, making bacterial UTIs the predominant form of the disease and the main indication for antimicrobial therapy (Foxman et al., 2000; Yang et al., 2022). Although many episodes resolve after standard treatment, recurrent urinary tract infections (rUTIs) represent a substantial clinical burden owing to their high frequency, repeated healthcare utilization, cumulative antimicrobial exposure and marked impact on quality of life (Hooton, 2012; Sze et al., 2025). Current clinical definitions of rUTIs are largely based on the temporal frequency of symptomatic episodes (Aydin et al., 2015; Anger et al., 2019). However, the terminology itself remains inconsistent, particularly in the distinction between recurrence, relapse, reinfection and persistence across studies and clinical settings (Dason et al., 2011; Aggarwal and Leslie, 2025; Hjelmager et al., 2025). These clinically pragmatic categories only partially reflect the biological heterogeneity underlying recurrent disease. Patients grouped under the same clinical label may differ substantially with respect to microbial persistence, intracellular or bladder wall-associated reservoirs, host immune response, anatomical predisposition, hormonal status and ecological context. At the same time, conventional diagnostic approaches continue to rely predominantly on symptoms and standard urine culture despite increasing evidence that these methods incompletely capture the microbial and functional diversity associated with recurrent disease (Josephs-Spaulding et al., 2021; Hochstedler et al., 2022). Standard urine culture, able to identify acute high-burden bacteriuria, is inherently biased toward fast-growing aerobic organisms. Recurrent infections may involve low-biomass communities, intracellular bacterial reservoirs, slow-growing or fastidious microorganisms, and polymicrobial ecological states that remain undetected by conventional protocols (Josephs-Spaulding et al., 2021; Perez-Carrasco et al., 2021; Hochstedler et al., 2022). Expanded quantitative urine culture, catheterized sampling, metagenomics and metabolomic profiling have consequently revealed far greater microbial complexity than previously recognized, including non-classical uropathogens, strain-level virulence adaptations and functional signatures associated with persistence and recurrence (Hochstedler et al., 2022; Neugent et al., 2022; Jiang et al., 2024; Yoo et al., 2024). These observations have progressively challenged the traditional view of rUTIs as repeated independent infections and instead support a more dynamic model involving host–microbe interactions across interconnected urinary, genital and intestinal ecosystems (Gilbert et al., 2017; Neugent et al., 2022; Timm et al., 2025). Nevertheless, despite these significant advances, a comprehensive understanding of rUTI pathogenesis remains elusive, reflecting both the dynamic regulation of bacterial virulence and the complex ecological landscape shaped by interactions among invading pathogens, the resident microbiota and host factors (Flores-Mireles et al., 2015; Josephs-Spaulding et al., 2021; Timm et al., 2025) (**Figure 1**). Collectively, these factors hinder a comprehensive understanding of the mechanisms driving colonization, persistence and recurrence. In this Review, we discuss how recent microbiological, immunological and multi-omic studies have reshaped current understanding of rUTIs, focusing on microbial persistence biology, urinary microbiota ecology, host susceptibility and emerging therapeutic strategies.

## 2 Recurrent urinary tract infections: not a simple definition

Recurrent urinary tract infections (rUTIs) are conventionally defined as two or more symptomatic episodes within six months, or three or more episodes within twelve months following complete clinical resolution of a previous infection (Dason et al., 2011; Aydin et al., 2015). Terminology remains inconsistent, particularly in the use of reinfection, relapse, persistence and recurrence. Although these terms are clinically useful, they should not be interpreted as direct evidence of the biological mechanism responsible for recurrence. The term “chronic UTI” is also problematic: it lacks a standardized definition, is not formally recognized in major guidelines, and is sometimes applied to non-infectious conditions such as interstitial cystitis/bladder pain syndrome (Homma et al., 2024; Hjelmager et al., 2025; Werneburg et al., 2026). This ambiguous terminology may affect diagnostic strategies, antimicrobial duration, further investigations, and interpretation of clinical and translational data (Gupta et al., 2001; Anger et al., 2019; Hjelmager et al., 2025). Emerging genomic evidence indicates that many recurrent episodes are biologically linked rather than entirely independent infections. Approximately half of recurrent UTIs are caused by the same bacterial strain as the initial infection. However, microbiological similarity alone does not establish the anatomical source of recurrence, as repeated episodes may reflect persistence within different host reservoirs, including the bladder, intestine, vagina, periurethral region or prostate (Timm et al., 2025). Despite being categorized as uncomplicated in many cases, traditional dichotomies such as uncomplicated versus complicated UTI offer pragmatic guidance but aggregate biologically heterogeneous scenarios under broad clinical labels. For example, UTIs in men were often classified by default as complicated, reflecting anatomical assumptions as opposed to a specific pathophysiology of the individual case. Although contemporary guidelines offer more nuanced frameworks, substantial variability persists across studies and specialties (Anger et al., 2019; Recurrent Uncomplicated Urinary Tract Infections in Women: AUA/CUA/SUFU Guideline (2025) - American Urological Association, 2025; EAU Guidelines, 2026) (**Table 1**).

**Table 1:** Summary of the principal clinical terms used to describe urinary tract infections (UTIs).

Term	Definition (concise)	Temporal pattern	Microbiological criteria	Key distinguishing features	Key references
Acute Urinary Tract Infection (UTI)	Acute-onset urinary symptoms associated with urinary inflammation and bacteriuria	Single, time-limited episode	Bacteriuria detected by urine culture; thresholds vary (≈10²–10^5^ CFU/mL depending on context)	Symptomatic infection involving the lower or upper urinary tract	(Hooton, 2012; Anger et al., 2019; Bonkat et al., 2025)
Uncomplicated UTI	Acute bacterial cystitis occurring in otherwise healthy, non-pregnant women without structural or functional urinary tract abnormalities	Episodic	Usually culture-positive; culture-negative symptomatic episodes recognized	Classic outpatient phenotype without major host risk factors	(Aydin et al., 2015; Anger et al., 2019; Bonkat et al., 2025)
Complicated UTI	UTI occurring in the presence of factors increasing risk of treatment failure or adverse outcomes	Variable	Often polymicrobial or associated with antimicrobial-resistant organisms	Broad and biologically heterogeneous category (obstruction, devices, immunocompromise, diabetes, renal disease, healthcare-associated)	(Hooton, 2012; Bonkat et al., 2025)
Localised UTI	Infection confined to the lower urinary tract without systemic signs	Episodic or recurrent	Bacteriuria may be present at variable thresholds	Preferred EAU terminology; outpatient management	(Bonkat et al., 2025)
Systemic UTI	UTI associated with systemic signs (e.g. fever, flank pain, bacteremia), with or without localized urinary symptoms	Acute, potentially severe	Often requires blood cultures and imaging	May require hospitalization and intravenous antibiotics	(Hooton, 2012; Bonkat et al., 2025)
Recurrent UTI	≥2 episodes within 6 months or ≥3 episodes within 12 months, with clinical resolution between episodes	Longitudinal, episodic	Episodes may be culture-positive or inconsistently documented	Clinically separated episodes separated by improvement; otherwise healthy patients	(Hooton, 2012; Aydin et al., 2015; Anger et al., 2019)
Reinfection	New symptomatic UTI after clinical resolution of a prior episode	>2 weeks after treatment or following documented eradication	Different organism, or the same organism following a sterile intervening culture	New infectious episode; implicit model underlying most rUTI definitions	(Dason et al., 2011; Anger et al., 2019)
Relapse	Early recurrence of UTI of the original infecting organism shortly after treatment following apparent eradication and a sterile urine culture	≤2 weeks after treatment	Same organism, often similar susceptibility profile	Protected bacterial reservoir or incomplete eradication	(Dason et al., 2011; Anger et al., 2019; Aggarwal and Leslie, 2025)
Persistent UTI	Continued bacteriuria and/or urinary symptoms despite appropriate antimicrobial therapy	>2 weeks after sensitivity-adjusted treatment	Same organism, ongoing bacterial growth without documented eradication	Reflects unresolved infection despite therapy; boundaries with relapse remain inconsistently defined; no standardized duration criteria	(Dason et al., 2011; Hjelmager et al., 2025)
Asymptomatic bacteriuria (ASB)	Presence of bacteriuria in the absence of urinary symptoms	Often chronic	≥10^5^ CFU/mL in defined specimens	Colonization rather than symptomatic infection; treatment generally discouraged	(Nicolle et al., 2005; Bonkat et al., 2025)
Chronic UTI	Non-standard term variably used to describe persistent or recurrent urinary symptoms attributed to infection	Ill-defined	Inconsistent or absent	Not formally recognized in major guidelines	(Hjelmager et al., 2025)
Chronic cystitis	Chronic inflammatory bladder symptoms of infectious or non-infectious origin	Chronic	Often culture-negative	Considerable overlap with non-infectious bladder syndromes	(Homma et al., 2024; Hjelmager et al., 2025)
Interstitial cystitis/bladder pain syndrome (IC/BPS)	Chronic bladder pain and urinary symptoms without proven infection	Chronic	No microbiological definition	Non-infectious; diagnosis of exclusion	(Homma et al., 2024; Werneburg et al., 2026)

UTI, urinary tract infection; CFU, colony-forming units; rUTI, recurrent urinary tract infection; ASB, asymptomatic bacteriuria; IC/BPS, interstitial cystitis/bladder pain syndrome.

## 3 Epidemiology across sex and age groups

Although national and regional level information is available to support effective public health policies, data on the global burden of UTIs remain limited. A recent comprehensive review estimated that UTIs affect more than 405 million individuals worldwide each year and are associated with approximately 240,000 deaths annually (Yang et al., 2022). Approximately 20-30% of UTIs are nosocomial, whereas the remainder are community-acquired (de Cueto et al., 2017). Women are the most frequently affected, with an estimated incidence of 80 cases per 1,000 population in 2019 (Yang et al., 2022), and 50–60% experiencing at least one UTI during their lifetime (Hooton, 2012; Sze et al., 2025). Following an initial infection, recurrence is common, occurring in approximately 20-30% of women within 6 months and rising to 44% within 1 year (Hekker et al., 2025). The epidemiological pattern varies across the female lifespan. In young sexually active women, UTI incidence reaches approximately 0.5-0.7 episodes per person-year (Foxman et al., 2000; Hooton, 2012; Hekker et al., 2025). In contrast, precise UTI incidence estimates in postmenopausal women are inconsistently reported. Nevertheless, UTIs represent one of the most common bacterial infections in this group and account for a substantial proportion of outpatient visits and hospitalisations, with recurrence rates increasing markedly after menopause, reaching up to 50% (Neugent et al., 2022; Hekker et al., 2025; Timm et al., 2025). In men, UTIs are less common, with an estimated incidence of approximately 22 cases per 1,000 population in 2019 (Yang et al., 2022), but the age-related increase is even more pronounced than in women. Lifetime risk is estimated at 13-15% (Bonkat et al., 2025; EAU Guidelines, 2026), with incidence rising from fewer than 3 per 1000 men younger than 55 years to nearly 8 per 1000 after 85 years (Caljouw et al., 2011; Schaeffer and Nicolle, 2016). Cohort data from male veterans indicate that 1.46% report at least one UTI annually, of which nearly 15% are recurrent (Ciudin et al., 2024; Drekonja et al., 2013). By seven years of age, 1.7% of boys and 8.4% of girls will have experienced at least one UTI (Hellström et al., 1991). Sex-related patterns differ during infancy: male infants younger than six months are approximately twice as likely as female infants to develop a UTI. However, among infants who have already experienced a UTI during the first year of life, recurrence rates appear comparable between sexes (32% in boys and 35% in girls) (Nuutinen and Uhari, 2001; Shaikh et al., 2008). Overall, one third to one half of affected children develop recurrent infections (Paintsil, 2013) (**Table 2**).

**Table 2:** Epidemiology of urinary tract infections (UTIs) and recurrent UTIs (rUTIs) across demographic subgroups.

Population	UTI epidemiology	rUTI epidemiology	Key references
Women (overall)	Lifetime risk: 50–60%	20–30% recur within 6 months; 44% within 1 year	(Foxman et al., 2000; Hooton, 2012; Sze et al., 2025)
Women (premenopausal)	Incidence: 0.5–0.7 episodes/person/year	No specific data	(Foxman et al., 2000; Hooton, 2012)
Women (postmenopausal)	No specific data	Recurrence up to 50%	(Neugent et al., 2022; Hekker et al., 2025; Timm et al., 2025)
Men (overall)	Lifetime risk: 13-15%	Around 15% of UTIs are recurrent	(Foxman, 2002; Ciudin et al., 2024; Bonkat et al., 2025)
Men <55 years	Incidence: <3/1000/year	No specific data	(Foxman, 2002)
Men >85 years	Incidence: 7-8/1000/year	Likely increased recurrence	(Caljouw et al., 2011; Schaeffer and Nicolle, 2016)
Children (overall)	By age 7: boys 1.7%, girls 8.4%	Recurrence 12-30%	(Hellström et al., 1991; Conway et al., 2007; Keren et al., 2015)
Infants (<6 months)	Higher incidence in males	Recurrence 32-35% after first UTI	(Nuutinen and Uhari, 2001; Shaikh et al., 2008)

## 4 Diagnostic and methodological blind spots

A microbiological diagnosis of UTI is conventionally based on compatible urinary symptoms associated with a positive standard urine culture (SUC), typically defined as ≥10^5^ CFU/mL (Bonkat et al., 2025; EAU Guidelines, 2026). Common symptoms include dysuria, urinary urgency and frequency, suprapubic pain, and haematuria. In rUTIs, however, this threshold may not be sufficiently sensitive, as symptomatic episodes can occur at significantly lower bacterial loads (Kaur and Kaur, 2021), with counts as low as 10²-10³ CFU/mL becoming clinically significant (Hilt et al., 2023). SUC is constrained by its methodology. Conventional protocols rely on small inoculum volumes, short incubation periods, and predominantly aerobic culture conditions (Brubaker et al., 2021; Josephs-Spaulding et al., 2021; Perez-Carrasco et al., 2021). Consequently, symptomatic patients may have negative standard cultures despite clinical evidence of infection, suggesting that conventional methods underestimate the microbial complexity of recurrent disease (Hooton, 2012; Brubaker et al., 2021; Josephs-Spaulding et al., 2021; Hochstedler et al., 2022; Neugent et al., 2022). Expanded quantitative urine culture (EQUC) overcomes these limitations by increasing urine volume, diversifying culture media, including anaerobic conditions, and prolonging incubation (Burnett et al., 2021; Perez-Carrasco et al., 2021; Shoemaker and Kim, 2021). In women with rUTIs, pathogen recovery has been reported in approximately 55% of catheterized samples, substantially exceeding SUC (Hochstedler et al., 2022). Culture-independent molecular approaches have further reshaped this paradigm. Bacterial 16S rRNA sequencing has demonstrated that both healthy individuals and patients with rUTIs harbor diverse bladder-associated microbial communities (Perez-Carrasco et al., 2021; Shoemaker and Kim, 2021). However, its limited resolution has led to increased use of shotgun metagenomics, which enables simultaneous detection of bacteria, fungi, and viruses, as well as characterization of virulence determinants and antimicrobial resistance genes (Brubaker et al., 2021; Josephs-Spaulding et al., 2021; Shoemaker and Kim, 2021). Compared with 16S rRNA sequencing, shotgun metagenomics also provides higher taxonomic resolution and, under appropriate analytical conditions, strain-level characterization (Simner et al., 2018; Chiu and Miller, 2019). Recent clinical studies have expanded the spectrum of microorganisms detected by shotgun metagenomics beyond conventional uropathogens, identifying organisms such as *Enterococcus*, *Klebsiella*, *Pseudomonas* and *Aerococcus*, as well as fungal and viral agents, particularly in polymicrobial or complicated infections (Jeff et al., 2025). However, these findings require careful clinical interpretation, as detection of microbial DNA alone cannot reliably distinguish active infection from colonization, clinically relevant organisms from background microbiota, viable microorganisms from residual DNA, or true bladder organisms from contaminants introduced during urine collection (Chiu and Miller, 2019; Jeff et al., 2025). A single metagenomic profile is therefore insufficient to establish the biological mechanism underlying a recurrent episode (Jeff et al., 2025). This expanding diagnostic landscape is consistent with systematic evidence showing that UTIs may also involve a broad range of atypical bacterial species (Gram-negative bacilli, Gram-positive cocci, diphtheroids, Mycoplasmataceae members, actinomycetes, and Gram-variable coccobacilli), as well as fungi, viruses, microsporidia, and Trich*omonas vaginalis*, particularly in selected clinical settings (Pereira de Lima et al., 2022). Biological interpretation therefore requires integration with standardized urine collection, complementary microbiological methods, longitudinal sampling across recurrent episodes, strain-resolved comparison where feasible, and the clinical context. These functional features provide biological information beyond taxonomic composition, but reflect genomic potential rather than confirmed microbial activity. Using shotgun metagenomics, Jiang et al. identified more than 790 virulence factor genes in women with active rUTIs, compared with 552 in controls, with enrichment of *Escherichia coli* O157:H7, *Klebsiella*, *Gardnerella*, and *Streptococcus* in this group of patients. The authors subsequently applied machine-learning models to predict rUTI disease status based on these virulence profiles (Jiang et al., 2024). In a complementary study, Neugent et al. linked metagenomic and metabolomic profiles to estrogen status, revealing enrichment of genes involved in lipopolysaccharide biosynthesis, iron acquisition, and antibiotic resistance in women with active or recurrent infections (Neugent et al., 2022). These metagenomic signatures describe the functional potential encoded within microbial genomes, rather than confirmed transcriptional activity during infection. Metatranscriptomic approaches can address this distinction by identifying genes that are actively expressed. Using targeted pangenome enrichment, Young et al. showed that gut *E. coli* from women with and without rUTIs displayed similar genomic gene content but distinct transcriptional programmes, highlighting biological differences that were not apparent from metagenomic gene presence alone (Young et al., 2024). These findings illustrate that microbial activity may reveal biological differences that are not apparent from gene presence alone. Another emerging approach is urinary cell-free DNA (cfDNA) sequencing, which integrates microbial detection with information on antimicrobial-resistance genes, bacterial growth dynamics, and host-response signals within a single assay. Its application in rUTIs remains preliminary, as available evidence derives predominantly from kidney transplant recipients and requires validation in broader rUTI populations before routine clinical implementation (Burnham et al., 2018; Liu et al., 2026). Despite their resolution, metagenomic approaches remain costly, technically demanding, and lack standardized analytical pipelines, limiting their routine clinical use (Perez-Carrasco et al., 2021; Shoemaker and Kim, 2021). Future clinical implementation will require methodological standardization and prospective clinical validation to define how these approaches can complement conventional microbiological diagnosis. Across studies, urine collection methods emerged as a further critical determinant of results. Midstream samples, although widely used, are prone to periurethral contamination, whereas catheterized specimens more accurately reflect bladder communities and provide more consistent microbial profiles (Burnett et al., 2021; Yoo et al., 2021; Hochstedler et al., 2022; Yoo et al., 2022; Yoo et al., 2024).

## 5 Risk factors and recurrence-prone hosts

Although shared biological mechanisms underlie recurrence, risk is shaped by distinct determinants across women, men, and children (Dason et al., 2011; Aydin et al., 2015; Anger et al., 2019; Hjelmager et al., 2025). Women represent the most affected population by rUTIs (Timm et al., 2025). Anatomical factors, including a short urethra and the proximity to vaginal and perianal microbial niches, facilitate ascending migration toward the bladder. In premenopausal women, recurrence is largely driven by behavioral and exposure-related factors, with sexual activity representing the strongest and most consistently identified risk factor. The frequency of sexual intercourse, new sexual partners, and the use of spermicides are all factors associated with an increased risk of recurrence (Foxman et al., 2000; Hooton, 2012). By contrast, in postmenopausal women, susceptibility reflects vaginal microbiome disruption, impaired bladder emptying, altered mucosal immunity, and estrogen depletion (Neugent et al., 2022; Dalby et al., 2025; Timm et al., 2025). In men, while a longer urethra provides partial protection from ascending exposure, recurrence is frequently driven by other structural abnormalities and bacterial reservoirs within the genitourinary tract. Bladder outlet obstruction, most commonly due to benign prostatic hyperplasia, promotes urinary stasis and incomplete emptying, creating conditions favourable for bacterial growth (Hooton, 2012). In addition, the prostate can act as a persistent reservoir, particularly in chronic bacterial prostatitis, where limited antibiotic penetration allows microorganisms to survive and intermittently reseed the bladder (Flores-Mireles et al., 2015; Schaeffer and Nicolle, 2016; Ciudin et al., 2024). Urinary tract calculi may also contribute to recurrence by acting as persistent bacterial reservoirs, particularly infection stones and struvite calculi, where microorganisms embedded within biofilms are protected from both host immunity and antimicrobial therapy, allowing intermittent reseeding of the urinary tract (Razi et al., 2024). Other contributors include urinary catheterisation and urological instrumentation, which disrupt mucosal barriers and facilitate bacterial entry, as well as advanced age, diabetes and neurological disorders that impair bladder function (Flores-Mireles et al., 2015). Androgen signalling may also contribute. Testosterone has been associated with increased uropathogenic *Escherichia coli* (UPEC) growth, endotoxin production, biofilm formation and epithelial invasion (Wu et al., 2024). In children, susceptibility is strongly influenced by developmental and anatomical factors that promote urinary stasis and bacterial persistence. Congenital anomalies of the kidney and urinary tract, including vesicoureteral reflux, obstructive uropathies, and duplicated collecting systems, are well established drivers of recurrence (Conway et al., 2007; Keren et al., 2015; Tewary and Narchi, 2015). Functional disorders, such as bladder-bowel dysfunction and dysfunctional elimination syndromes, further contribute through incomplete bladder emptying, urinary retention, and constipation, which favor bacterial growth (Tewary and Narchi, 2015). Infants younger than three months represent a particularly vulnerable group because of immune immaturity and the higher prevalence of structural urinary tract abnormalities that mature with aging. Recurrent infection is also associated with renal cortical scarring (15-65%), which is more strongly linked to recurrent episodes than to vesicoureteral reflux alone (Paintsil, 2013).

## 6 Microbial persistence biology

Although rUTIs are defined clinically over time, microbiological and genomic data indicate that many episodes are not independent infections. In a substantial proportion of cases, the infection involves the same bacterial strain, supporting a relapse model driven by microbial persistence, host susceptibility and ecological disturbance (Flores-Mireles et al., 2015; Kim et al., 2021; Sgarabotto et al., 2025). Recurrence depends on conserved virulence programs that enable stable colonisation of the urinary tract, including epithelial adhesion, nutrient acquisition, intracellular persistence, and biofilm formation. Additional protected niches may also contribute to bacterial persistence. Urinary calculi, particularly infection-related and struvite stones, represent biofilm-associated ecological microenvironments in which microorganisms become embedded within a complex mineral and organic matrix. These protected niches may facilitate long-term bacterial survival, contribute to recurrent infection, and promote intermittent reseeding of the urinary tract until complete stone clearance is achieved (Razi et al., 2024). Among uropathogens, UPEC remains the predominant etiological agent, accounting for approximately 75% of uncomplicated UTIs and 65% of complicated infections, and similarly dominating recurrent disease. UPEC can persist in periurethral reservoirs and repeatedly access the bladder, promoting both reinfection or relapse (Kim et al., 2021; Timm et al., 2025). Its adhesion is primarily mediated by fimbrial adhesins, such as type 1 fimbriae (FimH) and P fimbriae, which bind urothelial glycoprotein receptors and facilitate invasion of superficial umbrella cells (Josephs-Spaulding et al., 2021). In parallel, siderophore-mediated systems, including yersiniabactin-associated pathways, enable efficient iron acquisition within the nutritionally restricted urinary environment, supporting bacterial replication and persistence (Flores-Mireles et al., 2015; Timm et al., 2025). Following epithelial attachment, UPEC can invade urothelial cells and form intracellular bacterial communities (IBCs) that enable rapid replication while protecting bacteria from host immune responses and antimicrobial exposure. UPEC may also persist by acting as quiescent intracellular reservoirs (QIRs), consisting of metabolically inactive bacteria within deeper urothelial layers. In QIRs, bacteria can reactivate following epithelial turnover or inflammatory stimuli (Mysorekar and Hultgren, 2006; Kim et al., 2021; Sharma et al., 2021). Biofilm formation provides another persistence strategy across both intracellular and extracellular niches. Biofilms are structured microbial communities embedded in an extracellular polymeric matrix composed of polysaccharides, proteins, and extracellular DNA, which limits antimicrobial penetration and modulates host immune responses (Lila et al., 2023). Within these communities, metabolic heterogeneity promotes the emergence of persister cells, phenotypically tolerant subpopulations capable of surviving antibiotic exposure and reseeding infection after treatment cessation (Lila et al., 2023; Bhuiya et al., 2025). Phylogenetic background further contributes to recurrence biology. UPEC strains belonging to phylogenetic groups B2 and D are frequently enriched among recurrent isolates and are associated with increased virulence gene content and enhanced biofilm-forming capacity (Vautrin et al., 2023). However, these lineage-associated features do not define a unique recurrence-specific genotype, as comparative genomic analyses reveal substantial heterogeneity among recurrent isolates and no conserved recurrence-specific signature. A recent integrated ML–PanGWAS analysis further challenged the traditional view of recurrence as a lineage-driven phenomenon, showing that recurrent isolates do not segregate into distinct phylogenetic clades and that recurrence-associated signatures emerge only when chromosomal and plasmid-associated features are analysed together. Recurrent isolates were enriched for toxin–antitoxin systems, plasmid mobility-associated elements, stress-response pathways, and metabolic adaptation genes, supporting a distributed persistence phenotype across heterogeneous genomic backgrounds rather than a single recurrence-specific genotype (Rajendran et al., 2026). Recurrence therefore appears to reflect adaptive ecological fitness within the host, including point mutations, gene gain or loss, and mobilisation of plasmids and other mobile genetic elements that modulate adhesin expression, siderophore systems, stress response pathways, and antimicrobial tolerance (Maurizi et al., 2025). Persistence is not unique to UPEC. Several non-*Escherichia coli* uropathogens employ distinct strategies that support long-term survival within the urinary tract (Flores-Mireles et al., 2015)*. Klebsiella pneumoniae* combines multiple persistence strategies, including type 1 fimbriae-mediated urothelial adhesion, type 3 fimbriae-dependent biofilm formation, capsule-mediated immune evasion, and efficient siderophore-dependent iron acquisition. Its polysaccharide capsule, particularly in hypervirulent K1 and K2 serotypes, confers resistance to complement-mediated killing and phagocytosis, thereby promoting long-term persistence within the urinary tract (Schroll et al., 2010; Mim et al., 2026). *Enterococcus faecalis* relies on surface adhesins, including the enterococcal surface protein (Esp), together with pili and robust biofilm formation to establish persistent infection (Shankar et al., 2001; Sillanpää et al., 2010; Codelia-Anjum et al., 2023). Experimental and clinical studies have further demonstrated its ability to invade urothelial cells, providing an additional mechanism that may contribute to chronic and recurrent infection (Horsley et al., 2013, 2018). *Proteus mirabilis* persistence is closely linked to urease activity, biofilm formation, and infection stone development. Unlike UPEC, *P. mirabilis* rarely forms intracellular bacterial communities but instead establishes extracellular fimbria-dependent bacterial clusters that promote urinary stone formation and create protected ecological niches supporting recurrent and complicated UTIs (Schaffer et al., 2016; Yang et al., 2024). Although UPEC predominates, pathogen composition is not uniform across patient populations. In particular, postmenopausal women exhibit a broader and less *E. coli*-dominant spectrum, with increased representation of organisms such as *Klebsiella*, *Enterococcus*, and *Streptococcus*, consistent with epidemiological observations that a substantial proportion of infections in this group involve non-*E. coli* uropathogens and reflect underlying ecological instability (Hekker et al., 2025; Timm et al., 2025). In men, rUTIs are most commonly sustained by UPEC, although other *Enterobacteriaceae* and Gram-positive species, particularly *Enterococcus faecalis*, may also contribute, as they are frequently implicated in chronic bacterial prostatitis (Mendoza-Rodríguez et al., 2023). In children, rUTIs are typically caused by enteric Gram-negative bacteria, predominantly *E. coli*, but may include *Klebsiella*, *Enterobacter*, and *Pseudomonas* species in the presence of structural or functional abnormalities that favour persistence (Tewary and Narchi, 2015; Boulanger et al., 2025).

## 7 Urinary microbiota and cross-compartment ecology

The identification of a structured urinary microbiota has changed the interpretation of rUTIs. During the early phases of the Human Microbiome Project, the bladder microbiome was largely overlooked because uncontaminated sampling from healthy individuals required invasive procedures such as suprapubic aspiration (Turnbaugh et al., 2007). Advances in sequencing technologies and EQUC have demonstrated that urine contains a distinct and dynamic microbial ecosystem, influenced by sex, age, hormonal status, and host-factors (Kustrimovic et al., 2024). In healthy reproductive-age women, the most stable configuration is a *Lactobacillus* dominant urotype, particularly enriched in *Lactobacillus crispatus*, associated with low diversity and ecological dispersion. Other urotypes include *Lactobacillus iners*-dominated or mixed anaerobic profiles enriched in genera such as *Prevotella*, *Gardnerella*, *Atopobium*, *Corynebacterium*, and *Dialister* (Huang et al., 2022). At the phylum level, Firmicutes generally predominate, followed by Actinobacteria, Bacteroidetes, and Proteobacteria (Gottschick et al., 2017; Kustrimovic et al., 2024). Hormonal status strongly influences this ecology. Estrogen promotes epithelial glycogen accumulation and supports the growth of *Lactobacillus* species, maintaining a protective acidic environment. Estrogen deficiency, particularly in postmenopausal women, is associated with reduced *Lactobacillus* abundance and increased microbial diversity, conditions that favor colonization by Enterobacterales and other potential uropathogens (Gottschick et al., 2017; Dalby et al., 2025). Metataxonomic studies further refine this picture by highlighting the contribution of non-classical taxa. Huang et al. (2022) and Yoo et al. (2024) showed that women with rUTIs harbour more diverse urinary communities than healthy controls or women with acute sporadic cystitis, with enrichment of genera such as *Ralstonia*, *Prevotella*, *Dialister* and *Corynebacterium*. *Gardnerella* and *Atopobium* were recurrently identified in rUTI profiles, particularly when EQUC or sequencing was applied (Yoo et al., 2021; Huang et al., 2022). Vaginal dysbiosis characterized by reduced *Lactobacillus* and enrichment of *Gardnerella* may precede recurrent UPEC cystitis and facilitate reactivation of latent bladder reservoirs (Yoo et al., 2022). The pediatric urobiome that varies with age and sex, harbors genera including *Actinotignum*, *Corynebacterium*, *Streptococcus*, and *Escherichia* that may be detected even in asymptomatic individuals. Interestingly, children with multiple UTIs often exhibit decreased microbial diversity compared with those with fewer infections, suggesting ecological reduction associated with recurrent disease (Kelly et al., 2024). In men, communities are more heterogeneous and lack a consistent protective dominant taxon comparable to *Lactobacillus* in women. Communities may include *Corynebacterium*, *Streptococcus*, *Enterococcus*, *Prevotella*, and occasionally *Pseudomonas* (Groah et al., 2016; Modena et al., 2017; Ciudin et al., 2024). Strain-level analyses have demonstrated genetic overlap across interconnected microbial compartments. The gut is increasingly recognized as the principal reservoir of uropathogens that subsequently colonize the urinary tract through periurethral spread, defining the gut–bladder axis (Moreno et al., 2008; Czaja et al., 2009; Gilbert et al., 2017). More recent metagenomic studies further demonstrated that increased intestinal abundance of *Escherichia* and *Enterococcus* precedes bacteriuria caused by the corresponding organisms, with strain-level similarity between gut and urinary isolates (Magruder et al., 2019). In male rUTIs, overlap between urine and semen microbiota supports the role of the cited prostate-associated reservoirs contributing to recurrence (Groah et al., 2016; Ciudin et al., 2024). Microbial function is also relevant. Integrated metagenomic and metabolomic analyses link Enterobacterales-dominated communities to lipid-associated metabolites, whereas *Lactobacillus*-rich communities are associated with epithelial homeostasis and lower inflammatory signalling (Neugent et al., 2022). Microbial metabolites further modulate mucosal cytokine production and pathogen fitness. In particular, depletion of short chain fatty acid-producing taxa has been associated with altered immune tone and increased recurrence susceptibility (Neugent et al., 2025). Finally, therapeutic pressure contributes to ecological instability. Subinhibitory antibiotic exposure can induce transcriptional reprogramming in uropathogenic bacteria, enhance biofilm formation, and promote intracellular persistence, potentially impairing early mucosal clearance and perpetuating recurrence (Hoffman et al., 2005; Dalhoff, 2012; Goneau et al., 2014).

## 8 Immune imprinting and mucosal remodelling

The bladder urothelium represents the first and most critical interface at which host–microbe interactions determine whether bacterial exposure results in tolerance, transient infection, or progression to recurrence (Flores-Mireles et al., 2015). Far from being a passive barrier, the urothelium is an immunologically active tissue that integrates mechanical defense, innate immune sensing, and epithelial turnover during early infection (Timm et al., 2025). Alongside pathogen sensing, barrier integrity further determines susceptibility. The bladder luminal glycosaminoglycan layer (GAG) limits bacterial adhesion, and its disruption has been associated with rUTI and altered urobiome composition, particularly in postmenopausal women (Neugent et al., 2022). Urothelial cells express pattern-recognition receptors, including Toll-like receptors (TLRs), that detect microbial structures and initiate inflammatory signaling cascades (Sorić Hosman et al., 2022; Timm et al., 2025). Interindividual variability in these patterns contributes to rUTIs susceptibility, with polymorphisms in TLR2, TLR4, and TLR5 associated with altered bacterial signaling and increased rUTI risk in pediatric and adult cohorts, independent of vesicoureteral reflux (Karoly et al., 2007; Tabel et al., 2007; Hawn et al., 2009; Yin et al., 2010). Specifically, reduced TLR4 expression on peripheral monocytes has been observed in rUTIs-prone individuals compared with those experiencing sporadic infections, suggesting impaired early immune activation and bacterial clearance (Yin et al., 2010). Downstream TLR signaling further modulates infection outcomes. Interferon regulatory factors, particularly IRF3 and IRF7, calibrate the balance between antimicrobial defense and tissue-damaging inflammation. Genetic variants reducing IRF3 transcriptional activity have been linked to susceptibility to severe and recurrent infection, whereas reduced IRF7 expression may confer protection in specific clinical phenotypes (Fischer et al., 2010; Puthia et al., 2016; Sorić Hosman et al., 2022). In parallel, uropathogens actively subvert host responses; UPEC can dampen cytokine signaling and delay immune cell recruitment, facilitating epithelial invasion and intracellular persistence (Hunstad and Justice, 2010). At the effector level, impaired neutrophil recruitment, linked to reduced expression of IL-8 receptors (CXCR1 and CXCR2), alters neutrophil transmigration across the urothelium and has been implicated in rUTI-prone individuals (Frendéus et al., 2000; Smithson et al., 2005; Lundstedt et al., 2007; Sorić Hosman et al., 2022). Complementing these mechanisms, soluble innate immune effectors further reflect baseline defense competence by reflecting the balance between epithelial integrity and inflammatory activation. Tamm-Horsfall protein (THP - uromodulin) binds UPEC adhesins and limits colonization through glycan-dependent interactions; reduced urinary levels have been associated with rUTI and with tubular injury, whereas recovery correlates with clinical improvement (Rampoldi et al., 2011; Li et al., 2025). In contrast to THP, urinary levels of pentraxin 3 (PTX3), an inducible long pentraxin produced by immune and epithelial cells, are elevated during recurrent episodes and decreased with resolution (Bottazzi et al., 2010; Li et al., 2025). Moreover, susceptibility to rUTIs does not solely arise from insufficient immune activation. Exaggerated early inflammatory response, including elevated macrophage colony-stimulating factor (M-CSF) and prostaglandin E₂ (PGE₂), have been associated with subsequent recurrence, suggesting that excessive inflammation may promote urothelial damage and maladaptive remodelling (Hannan et al., 2010; Ebrahimzadeh et al., 2021; Sorić Hosman et al., 2022). On the other hand, insufficient innate activation permits prolonged bacteriuria and intracellular persistence (O’Brien et al., 2015). These findings suggest that protection requires a balanced response: sufficient immune activation to clear bacteria, but limited inflammation to avoid tissue damage (Sorić Hosman et al., 2022). Beyond the acute manifestations, infection can induce durable changes in urothelial structure and immune responsiveness. In a rat model of chronic intravesical *E. coli* infection, El-Mosalamy et al. observed progressive urothelial inflammation, hyperplasia, and dysplasia associated with sustained NF-κB activation, increased IL-6 expression, enhanced Bcl-2 signaling, persistent inflammatory cell infiltration, and increased urothelial proliferation. Lipopolysaccharide-mediated activation of TLR4/CD14 pathways promoted sustained NF-κB nuclear translocation and downstream inflammatory gene expression, while prolonged oxidative and nitrosative stress was linked to DNA injury and mutagenic remodeling within chronically inflamed urothelial tissues (El-Mosalamy et al., 2012). Intracellular bacterial persistence may further amplify these processes through stable epigenetic reprogramming. Using an *in vitro* model of chronic UPEC infection in human uroepithelial cells, Tolg et al. demonstrated increased DNMT activity and DNMT1 expression together with hypermethylation-mediated silencing of the tumor suppressor gene *CDKN2A* (*p16INK4A*). Chronic intracellular infection was also associated with downregulation of the DNA repair gene *MGMT*, linking persistent bacterial colonization to altered cell-cycle regulation, impaired genomic stability, and sustained epigenetic remodeling of infected urothelial cells (Tolg et al., 2011). At a broader ecological level, a recent review linked chronic urinary dysbiosis to persistent inflammatory and carcinogenic remodeling of the bladder microenvironment. Microbial metabolites, including reactive oxygen species, nitrogen-derived compounds, and other genotoxic products, were discussed as potential drivers of oxidative DNA injury, mitochondrial dysfunction, and persistent inflammatory activation within urothelial tissues. Extracellular matrix remodeling, epithelial–mesenchymal transition signaling, angiogenic pathways, and biofilm-associated persistence were further linked to chronic mucosal inflammation and repeated cycles of urothelial injury and repair (Trăn et al., 2025). Epidemiologic studies suggest that rUTIs and chronic cystitis are associated with an increased risk of bladder cancer recurrence and progression, with pooled estimates reaching an OR of 2.33 and an RR of 2.88, underscoring the clinical relevance of infection-mediated pathways (Akhtar et al., 2018). Persistent bacterial colonization may sustain chronic inflammation, promoting tissue remodeling and creating a microenvironment that favors tumor persistence and recurrence. Conversely, controlled activation of related innate immune pathways underlies the efficacy of intravesical Bacillus Calmette–Guérin (BCG) immunotherapy in non-muscle-invasive bladder cancer (Morales et al., 1976; Redelman-Sidi et al., 2014; Pan et al., 2025). Host age further modulates these responses. Immunosenescence is associated with impaired innate and adaptive immune function, reduced pathogen clearance, and altered inflammatory regulation, factors that may favor microbial persistence and increase susceptibility to recurrent infection (Siegel et al., 2020; Zeng et al., 2020; Conway and A Duggal, 2021). Experimental models further suggest that prior infection can durably reprogram urothelial differentiation and inflammatory responsiveness, generating a persistent “infection memory” that lowers the threshold for symptomatic recurrence upon re-exposure (O’Brien et al., 2015; Timm et al., 2025). This process includes infection-history-dependent rewiring of cytokine dynamics, such as altered TNFα signaling, which may enhance early bacterial control but predispose to chronic inflammation if sustained (Yu et al., 2019). Other immune compartments are also involved. Mucosal-associated invariant T (MAIT) cells provide rapid antibacterial surveillance but exhibit impaired functional responses in rUTI patients despite preserved cell numbers (Terpstra et al., 2020). In parallel, local adaptive memory with tissue-resident memory T cells (T_RM) can mediate localized protection following infection, although their development depends on sufficient antigen exposure and may be disrupted by early antibiotic treatment (Rousseau et al., 2023).

## 9 Therapeutic implications: from eradication to ecosystem modulation

Treatment of rUTIs is moving beyond episodic bacterial eradication toward strategies to target persistence, dysbiosis and cross-compartment reservoirs. Antibiotics remain the cornerstone of acute management, with short-course regimens targeting common uropathogens, particularly UPEC. In patients with a positive history of rUTIs, continuous low-dose prophylaxis, postcoital prophylaxis, and patient-initiated regimens can substantially reduce recurrence frequency during active treatment (Skuhala et al., 2025). However, repeated antibiotic exposure exerts strong selective pressure across intestinal, vaginal, and urinary microbiota, promoting expansion of resistant organisms and facilitating horizontal transfer of resistance determinants carried by plasmids and other mobile genetic elements (Sze et al., 2025). Recent real-world evidence suggests that, although long-term prophylaxis was not associated with increased hospitalization for resistant infections, it significantly increased the likelihood of isolating multidrug-resistant organisms, potentially limiting future therapeutic options and increasing reliance on broader-spectrum antibiotics (Sanyaolu et al., 2026). These limitations support strategies aimed at restoring protective microbial ecosystems. This is particularly evident in the vaginal compartment, where *Lactobacillus*-dominated communities maintain acidic pH, produce antimicrobial metabolites, and limit periurethral pathogen expansion. Antibiotic-induced depletion of these protective taxa may facilitate recolonization by enteric uropathogens and ascending reinfection (Akgül and Karakan, 2018; Gupta et al., 2024). In postmenopausal women, topical vaginal estrogen supports epithelial integrity and promotes *Lactobacillus* recovery, reducing recurrence risk (Moriconi et al., 2026). Beyond reducing recurrence, observational data from a large healthcare database associated vaginal estrogen prescription with lower risks of sepsis, hospitalization, and mortality in women with recurrent UTIs (LaClair et al., 2026). In this context of antibiotic conservation, methenamine hippurate is also being reconsidered as a non-antibiotic option for long-term prophylaxis (Heltveit-Olsen et al., 2025). Host immune modulation represents a complementary strategy. Sublingual polyvalent bacterial vaccines such as MV140 have demonstrated reductions in recurrence frequency and antibiotic use in selected cohorts, suggesting that mucosal immune training may enhance resistance to repeated pathogen exposure (Lorenzo-Gómez et al., 2022; Nickel and Doiron, 2023). Other interventions are more phenotype-dependent. Cranberry-derived proanthocyanidins may inhibit UPEC fimbrial adhesion, limited in patients with structural or neurogenic dysfunction (Williams et al., 2023; Jangid et al., 2025). Behavioural measures, including increased fluid intake, postcoital voiding, and avoidance of spermicides, remain relevant adjuncts in exposure-drive recurrence phenotypes (Corrales-Acosta et al., 2025). Nevertheless, a major unresolved therapeutic challenge remains the persistence of bacteria within protected bladder niches. IBCs and QIRs remain largely inaccessible to conventional therapies and may reactivate after treatment, providing a biological basis for relapse (Mysorekar and Hultgren, 2006; Flores-Mireles et al., 2015). Similar patterns have been observed in antibiotic-recalcitrant disease, where tissue-resident bacterial aggregates and biofilm-like communities persist within the urothelium and suburothelial compartments despite repeated antimicrobial exposure. Relapse risk appears to correlate more strongly with total bladder wall bacterial burden than with luminal microbial findings or *E. coli* detection alone (Gadhvi et al., 2025). All the observations support the development of reservoir-targeting therapies. In particular, bacteriophage-based approaches may be especially relevant in recurrent and multidrug-resistant phenotypes characterized by biofilm formation, chronic catheter-associated infection, or persistence within anatomically protected reservoirs. Unlike conventional broad-spectrum antibiotics, bacteriophages selectively target bacterial populations while largely preserving the surrounding commensal microbiota, and experimental evidence suggests that phage cocktails may enhance biofilm disruption and improve bacterial clearance when combined with antibiotics (Zalewska-Piątek and Nagórka, 2025). Nanotechnology-based intracellular delivery systems are emerging as potential strategies to overcome the limited intracellular penetration of conventional antibiotics in recurrent and refractory UTIs. Experimental platforms based on gentamicin-loaded nanogels and cell-penetrating peptide-conjugated carriers have demonstrated enhanced urothelial penetration, increased intracellular antibiotic uptake, and substantial clearance of intracellular bacterial communities in preclinical models. In particular, peptide-conjugated nanogels achieved approximately 36% higher intracellular gentamicin delivery compared with free drug exposure and promoted >90% bacterial clearance in murine models of intracellular *Pseudomonas aeruginosa* infection, supporting their potential role in targeting persistent reservoir-driven phenotypes resistant to standard therapies (Forte et al., 2024; Escobedo et al., 2025).

## 10 Limitations

The heterogeneity that emerged at multiple levels throughout this review reflects the difficulty in comparing the available studies. Starting from the epidemiological perspective, robust pathogen-specific data remain limited, likely owing to variability in surveillance systems, diagnostic practices, and case definitions. Likewise, current clinical definitions remain largely pragmatic and fail to capture the biological diversity underlying recurrence, including reinfection, intracellular persistence, reservoir-driven relapse, and chronic culture-negative phenotypes. In recurrence, the disease appears to correlate more closely with bladder wall-embedded bacterial reservoirs than with luminal microbial composition. Consequently, the diagnostic techniques most commonly used in both clinical practice and research have proven inadequate for pathogen detection, largely because of the underlying biological mechanisms. This contributes to diagnostic uncertainty and to the frequent discrepancy between persistent symptoms and negative urine cultures. Furthermore, the field still lacks a truly integrative and longitudinal framework capable of linking functional, metabolic, and strain-level microbial interactions with disease causality.

## 11 Conclusions

rUTIs remain difficult to prevent and treat because recurrence does not arise from a single mechanism. Across patient populations, recurrent episodes may result from reinfection, persistent bacterial reservoirs, impaired urinary clearance, microbial dysbiosis or altered host responses. Recent microbiological and multi-omic studies have expanded the understanding of rUTI pathophysiology beyond conventional culture-based diagnosis, highlighting mechanisms such as bladder persistence, cross-compartment microbial interactions and the influence of host context. At the same time, these findings underscore the limitations of symptom-based definitions and standard diagnostic approaches. Recurrent disease should not always be viewed as a process confined to the urinary tract itself. In clinical practice, recurrence often reflects the combined contribution of intestinal and genitourinary reservoirs, anatomical or functional abnormalities, impaired bladder emptying and host susceptibility. In these situations, repeated antimicrobial treatment alone is often insufficient, and long-term control may depend on identifying and addressing the factors that allow persistence or repeated reseeding of the urinary tract. A better understanding of the biological mechanisms underlying recurrence may help explain the heterogeneity observed among patients and lay the foundation for future possible personalized diagnostic and therapeutic strategies.
